# O049: Can the search-and-isolate strategy for controlling the spread of highly resistant bacteria (HRB) be mitigated?

**DOI:** 10.1186/2047-2994-2-S1-O49

**Published:** 2013-06-20

**Authors:** G Birgand, I Lolom, L Armand-Lefevre, S Belorgey, E Ruppé, A Andremont, J-C Lucet

**Affiliations:** 1 Infection control unit, Claude Bernard Hospital, Paris, France; 2 Bacteriology unit, Claude Bernard Hospital, Paris, France; 3 Bichat, Claude Bernard Hospital, Paris, France

## Introduction

French recommendations for controlling HRB spread (carbapenemase producing enterobacteriacae [CPE] and vancomycin resistant enterococci [VRE]) advocate an aggressive search-and-isolate strategy.

## Objectives

We describe the experience of a teaching hospital, adjusting control measures to the epidemiological risk analysis (EpiRA).

## Methods

From 01/2009 to 12/2012, 31 episodes (13 VRE, 16 CPE, 2 VRE+CPE [13 OXA-48, 3 KPC, 2 NDM1]) have been identified. An EpiRA was performed : time from admission to HRB+, number of cases, compliance with standard (SP) and contact precautions (CP), workload (of the index case (IC), of the ward), nurse-to-patient (Pt) ratio, antibiotic use. Measures were adapted to the EpiR assessment, from strict CP for a single case to cohorting with dedicated staff for carriers, contact Pts and newly admitted Pts (3 distinct areas) when secondary cases (SeC) were identified. Initial and weekly screening of contact Pts was systematically performed.

## Results

Pts were initially hospitalised in intensive care (n=8), medical (n=18) or surgical units (n=5). Length of stay varied from 3 days to 13 month (median, 20 days). 14 IC were identified within 48 h. following hospital admission. Strict CP were started for all IC, transfers and new admissions were stopped in 9/31 episodes, and after the occurrence of SeC for 5 others. The nursing staff was reinforced in 6 episodes and carriers were cared by dedicated staff in 3 other episodes, one with 3 distinct areas. SeC were identified in 9/31 episodes (7 VRE and 2 CPE), with 1 to 5 SeC/episode (18 SeC). 4 VRE IC identified 7 to 60 days after hospital admission generated 1 to 5 SeC (n=12, 3/episode); 3 VRE episodes with strict CP started at hospital admission generated 4 SeC (1.3/episode). 2 SeC were identified in 2 CPE episodes, 1 after 31 days and 1 after 72 days.

## Conclusion

This experience is the largest reported in France. SeC were more frequent in VRE (16 in 15 episodes) than in CPE (2 in 18 episodes). Our data suggest that control measures could be adapted according to EpiRA, if several conditions are gathered. However, SeC occurred around Pts under strict CP, highlighting that this strategy should be used prudently.

## Disclosure of interest

None declared.

